# Thrombocytopenia on the first day of emergency department visit predicts higher risk of acute kidney injury among elderly patients

**DOI:** 10.1186/s13049-017-0355-3

**Published:** 2017-02-10

**Authors:** Chia-Ter Chao, Hung-Bin Tsai, Chih-Kang Chiang, Jenq-Wen Huang, COGENT (COhort of GEriatric Nephrology in NTUH) study group

**Affiliations:** 1 Department of Medicine, National Taiwan University Hospital Jinshan Branch, New Taipei City, Taiwan; 2 Graduate Institute of Toxicology, National Taiwan University College of Medicine, Taipei, Taiwan; 3 Division of Nephrology, Department of Internal Medicine, National Taiwan University Hospital, 7 Chung-Shan South Road, Taipei, 100 Taiwan; 4 Department of Traumatology, National Taiwan University Hospital, Taipei, Taiwan; 5 Department of Integrative Diagnostics and Therapeutics, National Taiwan University Hospital, Taipei, Taiwan

**Keywords:** Acute kidney injury, Elderly, Emergency department, Geriatrics, Platelets, Thrombocytopenia

## Abstract

**Background:**

Few studies have addressed risk factors for acute kidney injury (AKI) in geriatric patients. We investigated whether thrombocytopenia was a risk factor for AKI in geriatric patients with medical illnesses.

**Methods:**

A prospective cohort study was conducted, by recruiting elderly (≥65 years) patients who visited the emergency department (ED) for medical illnesses during 2014. They all received hemogram for platelet count determination, and were stratified according to the presence of thrombocytopenia (platelets, <150 K/μL) during their initial ED evaluation. They were prospectively followed up during their ED stay. We analyzed the relationship between the diagnosis of thrombocytopenia and subsequent AKI after ED stay, using Cox proportional hazard modeling, with platelet count as a continuous variable or thrombocytopenia as a categorical variable.

**Results:**

Of 136 elderly patients (mean age of 80.7 ± 8.2 years, 40% with chronic kidney disease, and 39% with diabetes) enrolled, 22.8% presented with thrombocytopenia, without differences in baseline renal function. After a mean ED stay of 4.4 ± 2.1 days, 41.9% developed AKI (52.6% Kidney Disease Improving Global Outcomes [KDIGO] grade 1, 24.6% grade 2, and 22.8% grade 3). Patients with higher AKI severity had stepwise lower platelet counts compared to those without AKI. The Cox proportional hazard model revealed that lower platelet count as a continuous variable (hazard ratio [HR] 0.984, 95% confidence interval [CI] 0.975–0.994) and as a categorical variable (presence of thrombocytopenia) (HR 1.86, 95% CI 1.06–3.27) increased the risk of AKI. The sensitivity analyses accounting for nephrotoxic medications use, including non-steroidal anti-inflammatory drugs, vancomycin, and contrast, yielded similar results.

**Discussion:**

Thrombocytopenia is common among ED-visiting elderly, and the potential relationship between platelet counts and the risk of AKI suggests the utility of checking hemogram for those at-risk ofdeveloping adverse renal events.

**Conclusion:**

Thrombocytopenia on initial presentation might indicate an increased risk of AKI among elderly patients with medical illnesses.

## Background

Acute kidney injury (AKI), defined as a rapid decline in renal function, constitutes a silent epidemic and preferentially affects patients of advanced age. The incidence of AKI increase progressively with age, and the prevalence during hospitalization ranges from 10–11% among those aged 66–69 years to 30–35% among those aged >85 years, indicating a significant higher risk in the elderly than that among the general population [1]. The susceptibility of the elderly to acute renal insults might stem from physiologic and pathologic renal degeneration, as well as the impaired recovery ability of their kidneys [2]. Hospital-acquired AKI in elderly patients increases the risk of mortality by 3 to 6 fold compared to those without AKI, with mortality rates as high as 30 to 80% [3, 4]. In addition to mortality, AKI episodes in the elderly also increase the probability of a prolonged hospital stay, in-hospital complications, worsening functional status, and the chance of subsequent institutionalization [5, 6].

Due to the perceived importance of AKI, the risk factors that can precipitate such episodes have been widely investigated, but comparatively few studies have evaluated the risk factors for AKI in the elderly. In the Cardiovascular Health Study (CHS), researchers determined that patient demographics and comorbidities, such as advanced age, the presence of diabetes mellitus (DM) and chronic kidney disease (CKD), and pre-existing or subclinical vascular diseases might be associated with a higher risk of incident AKI among the community-dwelling elderly [7]. Other researchers have demonstrated that use of certain medications, such as antibiotics (vancomycin, aminoglycosides) and non-steroidal anti-inflammatory drugs, was associated with increased AKI events among elderly patients with critical illnesses [8]. However, using laboratory parameters to gauge the risk of subsequent AKI has not been studied widely in the geriatric population. Brown et al. reported that an elevated serum fibroblast growth factor 23 level predicted a higher risk of AKI in the elderly [9]. Results from CHS sub-analysis also indicated that blood-based inflammatory markers, including a higher baseline C-reactive protein, fibrinogen, and leukocyte counts were independently associated with an increased frequency of AKI episodes in the elderly [7].

Thrombocytopenia, a common finding among hospitalized patients, can result from infection, bone marrow suppression, consumptive processes, or treatment-induced complications including medications, surgery, or extracorporeal circuitry [10]. The development of hospital-acquired thrombocytopenia is potentially associated with a higher risk of adverse outcomes [11]. In addition, several studies have reported that thrombocytopenia after invasive procedures can be an important risk factor for the subsequent development of AKI in post-operative patients [12, 13]. However, none of these studies evaluated whether the relationship between thrombocytopenia and the incident AKI exists among elderly patients, a population with a higher tendency to develop thrombocytopenia [14]. Using a prospectively recruited cohort of geriatric patients, we aimed to investigate whether elderly patients with a finding of thrombocytopenia on the first day of emergency department (ED) visit had a higher risk of subsequent AKI after their ED stay.

## Methods

### Study participant characterization

Elderly (defined as age ≥ 65 years) patients visiting the NTUH ED for the evaluation of medical illnesses were enrolled between Jan and May during 2014, based on a convenience sample of patients admitted to the general medical wards of NTUH. During the initial evaluation in the ED, clinical features including the patients’ demographic profiles (age and sex) and past comorbidities were clarified by interview with patients themselves or their caregivers if they had altered consciousness, according to the routine evaluation process in ED. CKD was diagnosed if the patients had an estimated glomerular filtration rate (eGFR) < 60 ml/min/1.73 m^2^, calculated using their pre-ED visit baseline serum creatinine (Scr) based on Chronic Kidney Disease Epidemiology Collaboration (CKD-EPI) formula [15]. Vital signs including systolic blood pressure (SBP), diastolic blood pressure (DBP), heart rate, respiratory rate, and body temperature were measured as a part of the routine clinical evaluation. All of the participants underwent blood tests for a complete blood count and serum biochemistry analysis immediately after evaluation in the ED, dictated by their caring staff. The ED physicians provided a preliminary diagnosis for each participant after analyzing their clinical history, comorbidities, and laboratory data. The preliminary diagnoses were divided into different categories including cardiovascular or cerebrovascular events, pulmonary diseases, gastrointestinal and hepatic diseases, renal-urological diseases, infection of unknown origin, and others, according to criteria used in previous studies [16–18]. We collected clinical data, comorbidities, the initial vital signs, and laboratory data during the first day in the ED visit for all participants, and classified the patients according to the presence of initial thrombocytopenia (defined as platelet count < 150 × 10^3^/μL) or not immediately after their ED visit. The elderly participants were prospectively followed up since the first day of ED visit after blood tests until the day of their ward admission.

### Acute kidney injury as the clinical outcome

The outcome of interest in this study was the development of AKI on the day the patients were admitted to the general medical wards [5]. None of the participants were diagnosed with AKI during their initial evaluation in the ED, through a careful history taking to exclude the absence of recent changes in urine output levels or acutely worsening edema prior to ED visit during interview. It has been reported that the presence of peripheral edema is strongly associated with higher AKI incidence [19], and a change in urine output is an established criterion of Kidney Disease Improving Global Outcomes (KDIGO) classification for AKI [20]. Whether checking serum creatinine was necessary after interview was left to the attending physicians’ discretion. All of the participants underwent a test of their Scr after ED stay when they were admitted to the general medical wards. The diagnosis of AKI was based upon the comparison between the patients’ admission and baseline Scr data, using the (KDIGO) criteria [20], which defines AKI as a rise in Scr rise of least 0.3 mg/dLwithin 48 h or a 50% increase from their baseline levels. Stage 1, 2, and 3 AKI were classified according to a Scr increase of 50 to 100%, 100 to 200%, and ≥ 200% or an increase of Scr to ≥ 4 mg/dL, or the initiation of renal replacement therapy, respectively [21]. The baseline Scr levels were obtained from pre-admission medical records obtained within months before their ED visits.

### Statistical analysis

We first tested whether the continuous variables described in this study conformed to normal distribution, using a Kolmogorov-Smirnov test. If variables were normally distributed, they were expressed in the format of the means ± standard deviations, and comparisons were made between groups using an independent *t*-test; if the variables did not conform to normal distribution, they were expressed as medians with 25% and 75% values in parenthesis, and comparisons were made using a Mann-Whitney *U* test. Categorical variables were treated in the format of event numbers with percentages in parentheses, and comparisons between groups were made using the chi-square test. After dividing the cohort according to whether they had thrombocytopenia in the ED or not, we compared the clinical features, comorbidities, vital signs, and laboratory data between the 2 groups. Platelet counts were analyzed according to the different AKI grades, and the correlation between platelet counts and AKI severity was assessed using the Pearson correlation coefficient. We determined the cumulative hazard of developing AKI over the period of the ED stay, using stepwise Cox proportional hazard regression analyses with backward variable selection, with the development of AKI as the dependent variable. Cumulative hazard curves over the study period were also constructed to ascertain this relationship. The assumption of proportionality for platelet counts in the Cox model was checked by calculating the correlation between Schoenfeld residuals and the follow-up duration. We used SPSS 18.0 software (SPSS Inc., Chicago, IL, USA) in this study to perform the statistical analysis. Two-sided *p*-values less than 0.05 were considered statistically significant.

## Results

During the study period, a total of 1673 patients (without age selection) were admitted to all medical wards (including general and specialty-based ones), and another 107 patients died before admission. We enrolled 136 elderly patients who were admitted to the general medical wards, with a mean age was 80.7 ± 8.2 years, half were male, and they had a Charlson comorbidity index (CCI) of 7 (6–9). More than 50% of the participants had hypertension, 40% had CKD, 39% had DM, and 26% had a diagnosis of a malignancy. Pulmonary disorders were the common preliminary diagnoses (46%) made by the ED physicians. The participants stayed in ED for an average of 4.5 (3–6) days; at the end of follow up, 57 (42%) elderly patients were diagnosed with AKI after ED stay, with a median Scr of 1.1 (0.7–1.9) mg/dL immediately on ward admission.

Among the study participants, 22.8% had thrombocytopenia on the first day of their ED visit. Platelet counts were found to be normally distributed, with a Kolmogorov-Smirnov test *p* value of 0.2. The elderly patients with initial thrombocytopenia had a mean platelet value of 102 ± 51 × 10^3^/μL, compared to those without (262 ± 82 × 10^3^/μL) (Table 1). No significant differences were observed between the elderly patients with and without initial thrombocytopenia in terms of their demographic features, most of their comorbidities, CCI, the ED physicians’ preliminary diagnoses, and their initial vital signs in ED. We found that the elderly patients who presented with thrombocytopenia were significantly less likely to have hypertension and had significantly lower leukocyte counts (Table 1). Scr on admission and the ED length of stay did not differ between the groups with and without thrombocytopenia. In addition, the amount of fluid administration and the percentage of patients receiving nephrotoxic medications (non-steroidal anti-inflammatory agents, vancomycin, and others) did not differ between patients with and without thrombocytopenia.

**Table 1:** Elderly patients visiting emergency department for medical illnesses during the study period

Characteristics	Total	With thrombocytopenia	Without thrombocytopenia	*p* value
Demographic features				
Age (years)	80.7 ± 8.2	78.5 ± 9.1	81.3 ± 7.8	0.1
Gender (male %)	68 (50)	16 (52)	52 (50)	0.84
Comorbidities				
Diabetes mellitus (%)	53 (39)	14 (45)	39 (37)	0.43
Hypertension (%)	78 (57)	11 (35)	67 (64)	<0.01
Heart failure (%)	23 (17)	4 (13)	19 (18)	0.5
Coronary artery disease (%)	11 (8)	2 (6)	9 (9)	0.71
Peripheral artery occlusive disease (%)	9 (7)	3 (10)	6 (6)	0.44
Chronic obstructive pulmonary disease (%)	15 (11)	2 (6)	13 (12)	0.36
Chronic kidney disease (%)	54 (40)	14 (45)	40 (38)	0.48
Previous or active malignancy (%)	35 (26)	8 (26)	27 (26)	0.99
Peptic ulcer disease (%)	12 (9)	2 (6)	10 (10)	0.6
Charlson comorbidity index	7 (6–9)	7 (5–9)	8 (6–9)	0.1
Initial impression (%)				0.23
Cardio- or cerebro-vascular events	11 (8)	3 (10)	8 (8)	
Pulmonary diseases	63 (46)	10 (32)	53 (50)	
Gastrointestinal and hepatic diseases	21 (15)	9 (29)	12 (11)	
Renal diseases	14 (10)	3 (10)	11 (10)	
Infection of unknown origin	11 (8)	3 (10)	8 (8)	
Miscellaneous	16 (12)	3 (10)	13 (12)	
First vital signs obtained on ED evaluation				
Systolic blood pressure (mmHg)	131 (110.3–153)	133 (101–146)	130 (112–154)	0.89
Diastolic blood pressure (mmHg)	73 (62–84)	71 (60–82)	73 (62–84)	0.73
Heart rate (/min)	96.7 ± 20.8	96.8 ± 27.1	96.7 ± 18.7	0.98
Body temperature (°C)	37.2 ± 1.2	37.2 ± 1.2	37.3 ± 1.1	0.77
Respiratory rate (/min)	20 (18–22)	18 (18–22)	20 (18–22)	0.88
First laboratory data on ED				
Leukocyte counts (K/μL)	11.4 (7.9–15.6)	8.2 (4.9–12.3)	12 (8.4–16.5)	< 0.01
Hemoglobin (g/dL)	11.4 (9.6–12.8)	11.3 (8.8–12.4)	11.6 (9.6–13.1)	0.47
Platelet counts (K/μL)	225 ± 102	102 ± 51	262 ± 82	< 0.01
Baseline eGFR (ml/min/1.73 m^2^)	70.3 (42.7–88)	68.9 (39.3–84.4)	70.3 (42.7–89.8)	0.46
Serum creatinine on ward admission (mg/dL)	1.1 (0.7–1.9)	1.5 (0.9–2.1)	1 (0.7–1.7)	0.1
ED length of stay (days)	4.5 (3–6)	4 (3–6)	5 (3–6)	0.64

Data are expressed as mean ± standard deviation or median with 25% and 75% quartiles for continuous variables, and number (percentage) for categorical variables

*Abbreviation*: *ED* emergency department, *eGFR* estimated glomerular filtration rate

Of 57 (41.9%) elderly patients who developed AKI after their ED stay; 52.6% were classified as grade 1, 24.6% as grade 2, and 22.8% as grade 3. We analyzed the relationship between the initial ED platelet counts and subsequent AKI after ED stay. The elderly patients who presented with thrombocytopenia had a significantly higher incidence of AKI than the non-thrombocytopenic patients (61% vs. 36%). The elderly patients who were diagnosed with AKI on admission also had significantly lower initial platelet counts than those without AKI (200 ± 100 vs. 243 ± 100 × 10^3^/μL), and a higher proportion of initial thrombocytopenia (33% vs. 15%). The patients with higher AKI severity also had stepwise lower platelet counts compared to the patients without AKI (non-AKI vs. KDIGO grade 1 vs. 2 vs. 3, 243 ± 101 vs. 200 ± 109 vs. 215 ± 106 vs. 187 ± 82 × 10^3^/μL). A significant correlation was also observed between the initial platelet counts and the KDIGO grades (*r* = -0.19, *p* = 0.03).

There were no significant differences between the elderly patients who were diagnosed with AKI after ED stay and those who were not, in terms of their demographic profile and most of their comorbidities (Table 2). Elderly patients who were diagnosed with AKI on admission had a significantly higher proportion of DM, CKD, and higher CCI than those without AKI. The elderly patients with AKI after ED stay were more likely to have a preliminary diagnosis of gastrointestinal/hepatic and renal disorders, and pulmonary disorders were more common among the patients without AKI. The elderly patients with AKI also had significantly lower initial SBP, DBP, and eGFR than those without AKI (Table 2).

**Table 2:** Comparison of elderly patients with and without AKI after emergency department stay

Characteristics	AKI after ED stay	No AKI after ED stay	*p* value
Demographic features			
Age (years)	79.6 ± 8.4	81.5 ± 7.9	0.19
Gender (male %)	33 (58)	35 (44)	0.12
Comorbidities			
Diabetes mellitus (%)	28 (49)	25 (32)	0.04
Hypertension (%)	28 (49)	50 (63)	0.1
Heart failure (%)	12 (21)	11 (14)	0.28
Coronary artery disease (%)	4 (7)	7 (9)	0.7
Peripheral artery occlusive disease (%)	3 (5)	6 (8)	0.59
Chronic obstructive pulmonary disease (%)	7 (12)	8 (10)	0.7
Chronic kidney disease (%)	31 (54)	23 (29)	< 0.01
Previous or active malignancy (%)	18 (32)	17 (22)	0.19
Peptic ulcer disease (%)	7 (12)	5 (6)	0.23
Charlson comorbidity index	8 (6–10)	7 (6–8)	0.02
Initial impression (%)			< 0.01
Cardio- or cerebro-vascular events	2 (4)	9 (11)	
Pulmonary diseases	23 (40)	40 (51)	
Gastrointestinal and hepatic diseases	13 (23)	8 (10)	
Renal diseases	11 (19)	3 (4)	
Infection of unknown origin	2 (4)	9 (11)	
Miscellaneous	6 (11)	10 (13)	
First vital signs obtained on ED evaluation			
Systolic blood pressure (mmHg)	114 (95–141)	144 (123–162)	< 0.01
Diastolic blood pressure (mmHg)	64 (54–75)	78 (69–90)	< 0.01
Heart rate (/min)	94.5 ± 23.3	98.3 ± 18.8	0.3
Body temperature (°C)	37 ± 1.3	37.4 ± 1.1	0.06
Respiratory rate (/min)	19 (18–21)	20 (18–22)	0.69
First laboratory data on ED			
Leukocyte counts (K/μL)	12.3 (7.7–16.2)	10.6 (8–14.6)	0.12
Hemoglobin (g/dL)	9,7 (7.9–12)	11.7 (10.5–13.1)	0.06
Platelet counts (K/μL)	200 ± 100	243 ± 100	0.01
Baseline eGFR (ml/min/1.73 m^2^)	58.8 (33.5–83.8)	76.5 (51.1–91)	< 0.01
Serum creatinine on ward admission (mg/dL)	1.9 (1.4–2.5)	0.8 (0.6–1.1)	< 0.01
ED length of stay (days)	4 (3–5)	5 (3–6)	0.19

Data are expressed as mean ± standard deviation or median with 25% and 75% quartiles for continuous variables, and number (percentage) for categorical variables

*Abbreviation*: *AKI* acute kidney injury, *ED* emergency department, *eGFR* estimated glomerular filtration rate

**Fig. 1:**
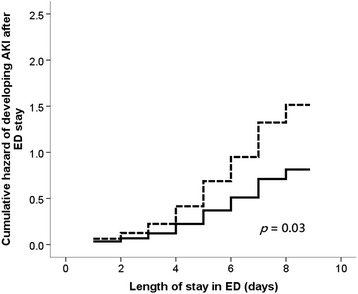
Cumulative hazard curve based on Cox proportional hazard regression analyses for evaluating the hazard of developing AKI after ED stay, based upon thrombocytopenia or not (solid line, without thrombocytopenia; dashed line, with thrombocytopenia). Abbreviation: AKI, acute kidney injury; ED, emergency department

**Fig. 2:**
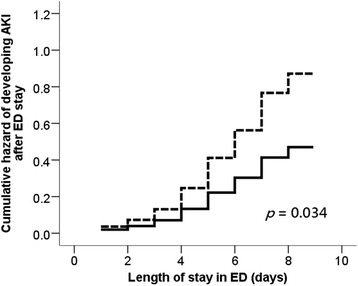
Cumulative hazard curve based on Cox proportional hazard regression analyses results of the sensitivity analyses (solid line, without thrombocytopenia; dashed line, with thrombocytopenia). Abbreviation: AKI, acute kidney injury; ED, emergency department

Using the Cox proportional hazard regression analyses, we constructed the cumulative hazard curves of AKI over the patients’ ED stay, based on elderly patients who presented with and without thrombocytopenia on the first day of ED visit (Fig. 1). We also used stepwise Cox proportional hazard regression analyses to delineate the relationship between initial thrombocytopenia or platelet counts on the day of ED visit and subsequent AKI after ED stay. The proportionality of platelet counts over the follow-up period was reassured by testing Schoenfeld residuals (*p* = 0.064). The stepwise Cox regression analyses revealed that lower initial platelet counts were predictive of a higher AKI risk (hazard ratio [HR] 0.997 per 10^3^/μL, 95% confidence interval [CI] = 0.994–0.999), and the presence of initial thrombocytopenia was similarly associated with a higher risk of subsequent AKI (HR 1.86, 95% CI = 1.06–3.27) among these patients (Table 3). The sensitivity analyses accounting for nephrotoxic medications use, including non-steroidal anti-inflammatory drugs, vancomycin, and contrast, disclosed that lower initial platelet counts were still predictive of a higher AKI risk (HR 0.997 per 10^3^/μL, 95% CI = 0.994–0.999), while thrombocytopenia was associated with a higher risk of subsequent AKI (HR 1.85, 95% CI = 1.05–3.28) (Table 3). The cumulative hazard curve constructed based on the sensitivity analyses showed similar findings (Fig. 2).

**Table 3:** Regression analyses with AKI after ED stay as the dependent variable

Cox proportional hazard regression	Hazard ratio	95% CI	*p* value
Model 1: PLT count as variable			
Comorbid CKD	1.88	1.11–3.2	0.02
Comorbid CHF	1.86	0.96–3.59	0.07
SBP at ED (per mmHg)	0.984	0.975–0.994	< 0.01
Platelet count (per 10^3^/μL)	0.997	0.994–0.999	0.02
Model 2: thrombocytopenia or not as variable			
Comorbid CKD	1.96	1.15–3.32	0.01
Comorbid CHF	1.96	1.01–3.83	0.048
SBP at ED (per mmHg)	0.984	0.974–0.993	< 0.01
Thrombocytopenia	1.86	1.06–3.27	0.03
^a^Sensitivity analyses: Model S1, PLT count as variable			
Comorbid CKD	1.77	1.01–3.09	0.04
Comorbid CHF	2.51	1.23–5.14	0.01
Comorbid malignancy	1.82	0.99–3.34	0.05
DBP at ED (per mmHg)	0.971	0.954–0.988	< 0.01
Platelet count (per 10^3^/μL)	0.997	0.994–0.999	0.01
^a^Sensitivity analyses: Model S2, thrombocytopenia or not as variable			
Comorbid CKD	1.99	1.16–3.39	0.01
Comorbid CHF	1.93	0.99–3.74	0.05
SBP at ED (per mmHg)	0.983	0.973–0.993	< 0.01
Thrombocytopenia	1.85	1.05–3.28	0.03

Model 1 and 2 included demographic features (age and gender), all comorbidities in Table 1, vital sign parameters (blood pressure and heart rates), and first laboratory data obtained (leukocytes, hemoglobin, and platelet counts)

^a^Sensitivity analysis models included demographic features (age and gender), all comorbidities in Table 1, vital sign parameters (blood pressure and heart rates), first laboratory data obtained (leukocytes, hemoglobin, and platelet counts), and use of NSAID, vancomycin, and contrast

*Abbreviations*: *AKI* acute kidney injury, *CI* confidence interval, *CKD* chronic kidney disease, *CHF* congestive heart failure, *DBP* diastolic blood pressure, *ED* emergency department, *NSAID* non-steroidal anti-inflammatory drug, *PLT* platelet, *SBP* systolic blood pressure

## Discussion

In the current study, we discovered that thrombocytopenia was common among elderly patients who visited the ED for medical illnesses; it occurred in nearly one-fourth of these medical encounters. After the ED stay, about 40% of the elderly patients were diagnosed with AKI, and those with subsequent AKI had more comorbidities as well as poorer renal function than the patients without AKI. The regression analyses that accounted for clinical features, initial diagnoses, and laboratory data revealed that the presence of thrombocytopenia on initial evaluation predicted nearly 2-fold higher risk of subsequent AKI, and the initial platelet count was a continuous variable that also exhibited a negative correlation with the subsequent risk of AKI. The sum of these findings suggests that checking a complete blood count can be a useful approach for estimating the risk of later AKI among elderly patients who present to the ED.

The prevalence of thrombocytopenia in this study fell within the range reported by other investigators. A decade-old study suggested that only 1% of all patients admitted to a general acute care hospital had thrombocytopenia [22]. However, as clinicians have increasingly recognized the importance of blood rheology, the frequency of complete blood count testing has risen, contributing to a higher incidence of thrombocytopenia being identified. Large scale studies have suggested that on average 8–36% of in-patients have thrombocytopenia, and the number increases further to 13–68% if they received specific medications (e.g., heparin), were admitted to intensive care units, or had higher illness severity [23, 24]. Since the current study focuses on geriatric patients with a mean age of 80.7 years, the percentage of patients visiting the ED with thrombocytopenia (22.8%) in this study seems reasonable. Similarly, the incidence of AKI in our geriatric cohort approximates the rates reported in the literature, since most of them suggest that the incidence falls between 30 and 40% among patients with extremely advanced age [1, 5, 6].

Prior studies have revealed that aberrant platelet levels, both thrombocytosis and thrombocytopenia, can be important outcome determinants among patients with episodes of acute inflammation or infection. Mirsaeidi et al. reported that admission platelet counts higher than 400 × 10^3^/μL or lower than 100 × 10^3^/μL predicted a higher 30-day mortality among patients with community-acquired pneumonia [25]. Prina et al. similarly indicated that the presence of thrombocytosis increased the risk of mortality and also the incidence of respiratory complications among patients with pneumonia [26]. In the Program to Improve Care in Acute Renal Disease (PICARD) study, the researchers discovered that thrombocytopenia early in the course of hospitalization also predicted a 60 to 110% higher risk of mortality among patients with critical illnesses [27]. However, studies linking thrombocytopenia and renal complications have been sparse and available reports illustrating the relationship between thrombocytopenia and AKI have mostly focused on patients undergoing invasive procedures. Van Linden et al. reported that the occurrence of thrombocytopenia in patients undergoing aortic valve implantation was associated with an increased AKI risk [28]. Aregger et al. also discovered a higher frequency of post-procedural thrombocytopenia as a significant risk factor for AKI after trans-aortic valve implantation [12]. We hypothesized that similar finding might exist for patients who did not undergo invasive interventions, and we observed that thrombocytopenia detected during the initial evaluation of patients with medical illness alone also predicted a higher risk of developing AKI (Table 3). These results lend support to our conclusion that thrombocytopenia is an important predictor of adverse outcomes, including not only higher mortality, but also an elevated risk of AKI. Our findings further suggest that this relationship applies similarly to the elderly, a population that is more susceptible to developing thrombocytopenia and AKI from clinical insults.

The participants in this study were somewhat older than the general population, with a high proportion having multimorbidity. This is related to our hypothesis that older adults are at higher risk of developing AKI, and we specifically enrolled those with age higher than 65 years. This advanced age might be reflective of the burden of population aging on the healthcare practice in our institute. In addition, increased platelet counts can be associated with inflammatory status. However, platelet counts are also affected by factors other than inflammation, including infection processes with platelet consumption, bone marrow reserve, and hepatic diseases [29]. Patients with hepatic disease (e.g. cirrhosis) or severe infection (e.g. sepsis) tend to develop leukopenia related to bone marrow suppression or splenic sequestration [30]. In Table 1, we observed a numerically higher percentage of thrombocytopenic patients presenting with gastrointestinal/hepatic diseases and infection of unknown origin than those without thrombocytopenia, although the difference did not reach statistical significance (*p* = 0.23). Consequently, we propose that thrombocytopenic patients in this study might exhibit a mixture of inflammation but a relatively higher prevalence of hepatic disease and/or infection than those without thrombocytopenia, leading to leukopenia (Table 1). The advanced age of these patients might partially underlie the exaggerated differences in leukocyte counts between thrombocytopenic patients and non-thrombocytopenic ones.

The rationale behind the relationship between initial thrombocytopenia and subsequent AKI is complex. Platelet count has been purported to be a marker for disease severity during acute illnesses of different nature, such as Plasmodium infection and Dengue fever [31, 32]. As inflammation/infection worsens, the consumption of platelet and thrombocytopenia may increase, and it may be compounded by local sequestration, disseminated intravascular coagulation and endothelial activation, leading to a further decline in platelet numbers [25]. An earlier presentation of thrombocytopenia during the disease course thus can be an occult indicator for ongoing inflammation, and persistent inflammation is a potential risk factor for AKI susceptibility [33]. In addition to their pro-coagulation and hemostatic effects, circulating platelets also exhibit immune-regulatory and anti-microbial ability by releasing peptides active against pathogens as well as through microorganism internalization [34]. Furthermore, an anecdotal report suggested that thrombocytopenia might directly contribute to the propagation of infection by lowering the secretion of transforming growth factor-β1 and altering cytokine repertoire [35]. Clinically, thrombocytopenia also confers a state of bleeding diathesis and increases the risk of major hemorrhage. There have been reports that thrombocytopenia elevated the risk of major bleeding complications by 4-fold among patients with acute myocardial infarction [36]. Severe bleeding with resultant hemodynamic instability is also a common predisposing event before hospital-acquired AKI. Finally, resuscitation for shock or massive fluid administration might cause dilutional thrombocytopenia, and such a clinical scenario is frequently accompanied by AKI [10, 37]. In our cohort, the predominant cause of AKI was ATN. In addition, we found that elderly patients who presented with thrombocytopenia had significantly lower leukocyte counts than the patients without thrombocytopenia (*p* < 0.01; Table 1), suggesting that a dilutional origin was another possible explanation. Although we did not measure inflammatory markers or assess fluid status among all the participants, we propose that thrombocytopenia might be an early marker for persistent inflammation or an excess volume status, leading to susceptibility for AKI, or it might even play a directly pathogenic role in immune-dysregulation, precipitating severe infections and resultant AKI. Based on our findings, it may be worthwhile to order an assay for platelet count among elderly patients presenting to ED for medical illnesses, for gauging their AKI risk. Avoidance of nephrotoxin exposure could also be considered for elderly patient who present to the ED with thrombocytopenia.

Our study has its strengths but is also limited in several aspects. The existence of a relationship between initial thrombocytopenia and the subsequent risk of AKI has only been reported among patients undergoing invasive procedures in the existing literature, and we confirmed the same connection in elderly patients with medical illnesses. However, the relatively modest number of cases, the absence of data about inflammatory markers, the single center study design, and the restriction of patients to those admitted to general medical wards only, reduce the generalizability of our findings. A competing risk from death during ED stay should also be considered when interpreting our results, although the mortality rate in our ED is relatively low. The lack of data on inflammatory markers and the absence of a fluid status assessment limit the interpretation of the relationship between thrombocytopenia and AKI observed in this study. In addition, depending upon clinical settings, the etiologies of AKI differ, and sepsis has been reported to be the leading cause of AKI [38]. In the current study, the possibility exists that the presence of sepsis partially accounts for the association between thrombocytopenia and the occurrence of AKI, although we have tried to adjust for this issue by considering relevant parameters including leukocyte counts and physiologic variables such as blood pressure and heart rate in the regression models. Furthermore, the extensive adjustment of known interfering variables and the existence of a dose-dependent relationship (higher AKI severity, greater decline in platelet count) strengthens the credibility of our results. Finally, results from our current approach might be influenced by the possibility that some participants would develop AKI but went unnoticed before they were admitted. A fixed time period model would be more appropriate in this regard. More studies are needed for validation.

## Conclusion

AKI constitutes an important concern for hospitalized patients, particularly those of advanced age, since the development of AKI increases their risk of adverse outcomes. Few studies have addressed the risk factors for AKI among geriatric patients, and none of the existing studies used thrombocytopenia or platelet counts as a predictive variable, despite its simplicity and wide availability. By prospectively recruiting elderly patients with medical illnesses who visited the ED, we discovered that thrombocytopenia detected during the initial evaluation significantly increased the risk of subsequent AKI, and the risk of AKI rose progressively with lower initial platelet counts. Checking the platelet level could be an informative approach for evaluating renal outcomes among elderly patients; it might be rational to avoid nephrotoxin exposure, optimize hemodynamic status, and provide early treatment of underlying diseases among thrombocytopenic elderly patients in the ED.

## Abbreviation

AKI: Acute kidney injury
CCI: Charlson comorbidity index
CHS: Cardiovascular Health Study
CI: Confidence Interval
CKD: Chronic kidney disease
CKD-EPI: Chronic Kidney Disease Epidemiology Collaboration
DBP: Diastolic blood pressure
DM: Diabetes mellitus
ED: Emergency department
eGFR: Estimated glomerular filtration rate
HR: Hazard ratio
KDIGO: Kidney Disease Improving Global Outcomes
NTUH: National Taiwan University Hospital
PICARD: Program to Improve Care in Acute Renal Disease
SBP: Systolic blood pressure
Scr: Serum creatinine
